# Thermal Death Point of *Baylisascaris procyonis* Eggs

**DOI:** 10.3201/eid1301.060966

**Published:** 2007-01

**Authors:** Shira C. Shafir, Wei Wang, Frank J. Sorvillo, Matthew E. Wise, Laurel Moore, Teresa Sorvillo, Mark L. Eberhard

**Affiliations:** * UCLA School of Public Health, Los Angeles, California, USA; † Los Angeles County Department of Health Services, Los Angeles, California, USA; ‡ Centers for Disease Control and Prevention, Atlanta, Georgia, USA

**Keywords:** Baylisascaris. procyonis, roundworms, thermal death point, neurologic, baylisascariasis, letter

**To the Editor:** In the past 20 years, *Baylisascaris procyonis*, the common intestinal roundworm of raccoons, has increasingly been recognized as a source of severe human neurologic disease that particularly affects children (*1*,*2*). Although human baylisascariasis appears to be rare, the devastating neurologic disease caused by this infection and the lack of effective treatment make it a disease of public health importance (*3*).

Adult raccoons infected with *B. procyonis* can shed millions of unembryonated eggs in feces daily (*4*). Once infective, eggs can remain viable in the environment for years and are resistant to most decontamination methods (*5*). Given the severe and untreatable nature of baylisascariasis, and the hardy nature of *B. procyonis* eggs, information on optimal methods to inactivate *B. procyonis* eggs is essential. To guide attempts at environmental decontamination as well as personal protection in the case of accidental or intentional contamination of drinking water supplies, we attempted to determine the thermal death point of *B. procyonis* eggs.

Experiments were conducted in which 150 μL each of embryonated eggs, at a concentration of 100 eggs per μL, were added to six 1-mL polypropylene tubes of sterile water. The 6 tubes were then added to a water bath at 35°C and allowed to sit for 10 min to equilibrate. Then the temperature of the water bath was slowly increased at a rate of ≈5°C per 7 min, and 1 tube was removed at each 5° increment from 37°C to 62°C. Eggs were then examined by light microscopy to determine whether the larvae were still viable, as judged by larval motility (Figure). The experiment was repeated by using a more objective assessment of viability through examination of hatched larvae. Inactivation was measured with a viability dye (methylene blue) exclusion method in which uptake of dye by larvae indicates cell death and inactivation. After the eggs were removed from the heat, the mammilated layer was removed through exposure to undiluted chlorine beach and then washed 5 times in 0.85% saline for 1 min at 600×*g*. Hatching was achieved by the glass bead method (*6*,*7*). Hatched larvae were then removed and mixed 1:1 with a 1:10,000 dilution of methylene blue. Viable larvae remained motile and had an intact cuticle that could not be penetrated by the stain, whereas nonviable larvae took up the methylene blue along the cuticle and stained blue (*8*).

**Figure:**
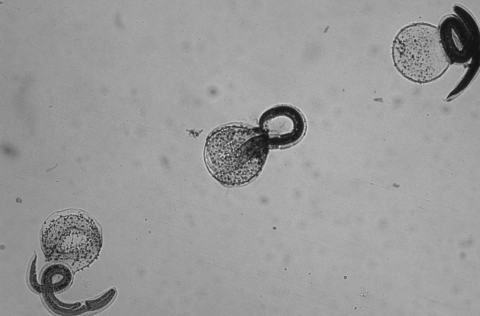
Hatched, stained, nonviable *Baylisascaris procyonis* larvae (magnification ×10).

The experiment was repeated by adding the heated water, in 5° increments between 37°C and 62°C, directly into the tube containing the eggs. The duration of exposure of the eggs to the water was <1 min. The eggs were then processed in the same manner as previously described and examined by light microscopy. All experiments were replicated.

All larvae remained viable in water up to 47°C; >75% of the larvae were viable at 52°C and 57°C; complete inactivation occurred at 62°C. When the heated water was added directly to the infectious eggs, all larvae remained viable up to 42°C, and most larvae were observed to be viable at 47°C and 52°C; complete inactivation occurred at 57°C.

These preliminary findings indicate that *B. procyonis* eggs have a thermal death point, <62°C, very similar to the thermal death point of *Ascaris lumbricoides* and *A. suum* (*9*). Given the widespread prevalence of *B. procyonis* in raccoons, the close association of raccoons with human populations, and the serious nature of infection, identification of the thermal death point of infectious *B. procyonis* larvae has important implications. Potential for human infection can be mitigated by decontaminating areas where *B. procyonis* eggs are known to be found. Health authorities and parasitologists are routinely contacted by citizens and organizations regarding concerns about areas that have been contaminated with raccoon feces, including yards, pools, and homes. Unfortunately, no comprehensive studies have been published that describe practical and effective methods for decontamination of areas where *B. procyonis* eggs are present. The recognition of complete inactivation of eggs at relatively low temperatures will provides guidance in circumstances in which natural or intentional contamination with *B. procyonis* eggs requires disinfection efforts and indicates that approaches well short of incineration or boiling will be effective. Furthermore, these results suggest that temperatures achievable in point-of-use hot water heaters (household units) can deactivate infectious *B. procyonis* eggs, thus providing an option for maintaining safe drinking water during a possible event of bioterrorism or a “boil water advisory.” However, further efforts are needed to determine the effectiveness of heat and other disinfection methods on inactivation of eggs in natural circumstances such as in feces or contaminated play areas including soil.
